# Disentangling rock record bias and common-cause from redundancy in the British fossil record

**DOI:** 10.1038/ncomms5818

**Published:** 2014-09-04

**Authors:** Alexander M. Dunhill, Bjarte Hannisdal, Michael J. Benton

**Affiliations:** 1 Department of Biology and Biochemistry, University of Bath, Claverton Down, Bath BA2 7AY, UK; 2 School of Earth Sciences, University of Bristol, Wills Memorial Building, Queen’s Road, Bristol BS8 1RJ, UK; 3 Centre for Geobiology, Department of Earth Science, University of Bergen, Allegaten 41, N-5007 Bergen, Norway; 4 Present address: School of Earth and Environment, University of Leeds, LS2 9JT, UK

## Abstract

The fossil record documents the history of life, but the reliability of that record has often been questioned. Spatiotemporal variability in sedimentary rock volume, sampling and research effort especially frustrates global-scale diversity reconstructions. Various proposals have been made to rectify palaeodiversity estimates using proxy measures for the availability and sampling of the rock record, but the validity of these approaches remains controversial. Targeting the rich fossil record of Great Britain as a highly detailed regional exemplar, our statistical analysis shows that marine outcrop area contains a signal useful for predicting changes in diversity, collections and formations, whereas terrestrial outcrop area contains a signal useful for predicting formations. In contrast, collection and formation counts are information redundant with fossil richness, characterized by symmetric, bidirectional information flow. If this is true, the widespread use of collection and formation counts as sampling proxies to correct the raw palaeodiversity data may be unwarranted.

## Abstract (web summary)

Palaeodiversity estimations are confounded by fossil availability and sampling. Here, Dunhill *et al.* use the fossil record of Great Britain to test aspects of the marine and terrestrial fossil records that are commonly used to identify and correct for bias in palaeodiversity estimations.

## Graphical abstract

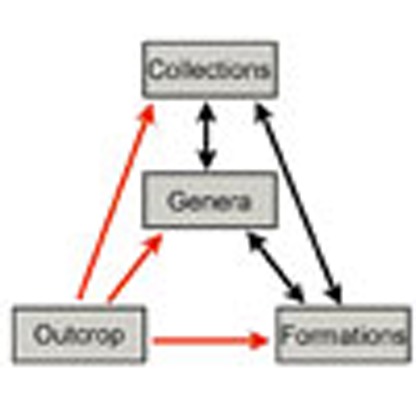

Understanding biotic evolution through deep time is a key research agenda in palaeobiology and Earth system science^1^,^2^,^3^. The evidence resides in the fossil record, and yet this record is compromised by incompleteness and bias, and it has been debated whether bias dominates the data^1^,^4^,^5^,^6^,^7^,^8^,^9^, or not^10^,^11^,^12^,^13^. Further, in the search for methods to provide a bias-free, or corrected, palaeodiversity signal, proposals have been made to use rock outcrop areas and collection and formation counts as sampling proxies to correct the raw palaeodiversity data^8^,^14^,^15^,^16^,^17^,^18^, but the usefulness of such approaches has been queried^19^,^20^.

There are further implications for interpreting how life diversified from a single species billions of years ago to 5–10 million eukaryotic species today^21^, and the consequent impact on global climate, and chemistry of oceans and atmosphere: do palaeodiversity records support a heavily damped and perturbed exponential model of diversification^22^,^23^,^24^, did biodiversity reach an equilibrium level equivalent to today’s biodiversity 500 Myr ago^4^,^6^, or has the diversity of individual clades fluctuated idiosyncratically in response to adaptive radiations and extinctions over the past 500 Myr^1^? The latter two hypotheses imply that the apparent rise in palaeodiversity through the past 500 Myr must be explained as sampling bias. It has not been clear how this debate could be resolved^1^,^4^,^6^,^10^,^12^ except by the input of alternative data sets or methods.

The most commonly implemented techniques to account for bias are sampling standardization, which seeks to equalize or make sampling fair at the level of collections^1^,^25^,^26^,^27^ and, model fitting using sampling proxies to identify times of poor and good sampling and to apply *post hoc* corrections^8^,^17^,^27^. Sampling proxies include measures of rock outcrop area, geological formations and collections.

Palaeodiversity (fossil taxonomic richness) curves and their covariation with sampling proxies can be explained by any of three hypotheses, alone or in combination: (1) the rock record bias (RRB) hypothesis^4^,^5^,^6^,^7^,^27^ that variability in the amount of available rock determines fossil diversity; (2) the common-cause (CC) hypothesis^11^,^13^,^28^,^29^,^30^ that much of the covariation of fossil and rock records is because both are driven by a third factor such as sea level change; or (3) the redundancy (RED) hypothesis^20^,^31^ that rock and fossil record proxies covary because of operational redundancy (that is, more collecting may result in greater richness, but high richness may also result in more collecting) and statistical redundancy (that is, various time series are different versions of the same signal), reflecting the mutual reinforcement of the sampling proxy and fossil richness. It is important to note that the CC and RED hypotheses are distinct in that the CC requires that two non-redundant variables are driven by a third variable, whereas the RED requires a two-way causality between variables without a third factor driving the dependence. The outstanding question is how to quantify the relative importance of these three, likely non-mutually exclusive, hypotheses. What is needed is some statistical means of indicating the directionality of potential drive–response relationships, in lieu of mechanistic process models.

Regional scale, rather than global, studies may provide a useful approach^32^,^33^,^34^,^35^, and if well chosen, they can offer the advantage of comprehensive, evenly reported data and focus on a single geological history. Here, we present an investigation of the rock and fossil records of Great Britain, which, despite being recorded from a relatively limited geographic region, encompass an almost continuous range of stratigraphic intervals that are probably the most intensely geologically sampled and documented area in the world^35^,^36^,^37^. Geological data have been recorded for over 200 years and the British Geological Survey has established detailed, fine-scale stratigraphies, and made its rich data stores available in electronic, georeferenced format (Fig. 1).

Here, we evaluate the RRB, CC and RED hypotheses using information transfer (IT), a non-parametric technique for quantifying the relative strength and directionality of predictive information flow between components of a coupled system (see Supplementary Methods and Supplementary Figs 1–5)^13^,^38^,^39^. We apply pairwise correlation and IT to detailed Phanerozoic marine and terrestrial stratigraphical data sets from Great Britain (Fig. 2), global environmental proxy (Supplementary Fig. 6), and palaeodiversity data (downloaded from the Paleobiology Database (PaleoDB; http://paleobiodb.org/; Fig. 2). Sampling proxies are assessed at the epoch level to determine how well they correlate with, and predict, palaeodiversity.

Under the RRB hypothesis, we expect rock quantity proxies (that is, formation counts and outcrop area) to have the strongest influence on palaeodiversity, beyond mutual correlations with other environmental variables, and we expect IT to flow from rock quantity to palaeodiversity. For the CC hypothesis, other environmental proxies would have the strongest influence on palaeodiversity, beyond mutual correlations with rock quantity, and IT would flow from environmental proxies to palaeodiversity. For the RED hypothesis, we expect sampling proxies whose origin is partly controlled by the distribution of fossil richness in the field (for example, collections and formations) to show strong, bidirectional IT with palaeodiversity, to be redundant with palaeodiversity in conditional IT (CIT), and to respond to the same environmental drivers as palaeodiversity. Because the three hypotheses are non-exclusive, and because each of the observed records may capture multiple underlying processes, the predicted relationship between pairs of variables may not be uniquely specified under each hypothesis, but may vary depending on the relationship between other variables. Assessing the relative degree of support for the different hypotheses therefore requires the full combination of statistical results.

We find some common signals, and some differences between the marine and terrestrial data. Both data sets show that collection and formation counts are information redundant with fossil richness, characterized by symmetric, bidirectional information flow. However, whereas marine outcrop area contains a signal useful for predicting changes in genera, collections, and formations, terrestrial outcrop area contains a signal useful for predicting only terrestrial formations.

## Results

### Marine data

In the marine data, the strongest correlations are found between palaeodiversity (genera) and collections, between genera and formations and between formations and outcrop (see Supplementary Table 1 for correlation coefficients). The correlation between genera and outcrop area is not significant after false discovery rate correction, and compared with genera, collections show weaker correlations with both formations and outcrop. It is important to note that correlations are calculated after first differencing, which isolates short-term (bin-to-bin) changes and filters out longer-term variation. In contrast, IT takes into account both short- and long-term variation, relying instead on surrogate data to accommodate for autocorrelation. IT analysis thus modifies and expands our view of the statistical relationships in the marine data (Fig. 3). There is strong, bidirectional IT between genera and collections (Fig. 3a), and although there is a slight asymmetry favouring IT from collections to genera, this asymmetry is very rarely significant (Collections>Genera is rarely detected), suggesting that they predict each other equally well. The IT between formations and genera is somewhat weaker, but also bidirectional (Fig. 3b). IT from genera to formations is detected with slightly higher frequency than in the opposite direction, but again this asymmetry is not significant. Collections and formations (Fig. 3c) show essentially the same pairwise IT association as genera and formations, the only notable difference being that (contrary to the correlations) the relationship between the former appears slightly stronger than between the latter. The IT between genera and outcrop area also converges on significance in both directions, but in this case there is significant asymmetry in favour of IT from outcrop to genera (Outcrop>Genera), suggesting that changes in outcrop area can be used to predict changes in palaeodiversity more than vice versa (Fig. 3d). IT from outcrop to collections shows the same significant directionality (Outcrop>Collections), albeit with a slightly lower detection rate (Fig. 3e). Finally, the IT between formations and outcrop area is the most clearly asymmetric relationship (Outcrop>Formations), verging on unidirectional, suggesting that outcrop is much more useful for predicting formations than vice versa (Fig. 3f).

These findings together imply that in the marine data, genera and collections are tightly linked to each other, and to a lesser extent to formations, through symmetric, bidirectional IT, while outcrop area contains a signal useful for predicting changes in genera, collections and formations (Fig. 4).

CIT analyses of the marine data suggest that formations contribute no information on genera not already provided by collections, and little information on collections not already found in genera (Fig. 5a). CIT from formations to collections is slightly stronger than from formations to genera (Fig. 5a), attributable to a slight asymmetry (typically insignificant) in the pairwise IT from collections to genera (Fig. 3a). Genera and collections have the strongest mutual relationship, but outcrop nonetheless provides significant CIT (Fig. 5b). Although pairwise IT from outcrop to collections is no greater than from outcrop to genera (Fig. 3d), CIT from outcrop to collections given genera exceeds CIT from outcrop to genera given collections (Fig. 5b), again attributed to pairwise asymmetry between collections and genera (Fig. 3a). Outcrop contains non-redundant CIT on both genera and formations (Fig. 5c). Pairwise IT from outcrop to formations (Fig. 3f) roughly equals that from outcrop to genera (Fig. 3d), but because pairwise IT from genera to formations is slightly (if insignificantly) greater than from formations to genera (Fig. 3b), conditioning on genera results in weaker CIT from outcrop to formations (Fig. 5c). Replacing genera with collections involve the same interrelationships, but pairwise IT from collections to formations (Fig. 3c) is slightly stronger than from genera to formations (Fig. 3b), resulting in even weaker CIT from outcrop to formations conditioned on collections (Fig. 5d) relative to conditioning on genera. Although both genera and collections show significant pairwise IT to outcrop (Fig. 3d,e), both are typically insignificant when conditioned on formations (Fig. 5e). The latter is even weaker, both in pairwise IT (Fig. 3f) and CIT (Fig. 5e). Any IT from genera and collections to formations or outcrop disappears when genera and collections are conditioned on each other (Fig. 5f).

### Terrestrial data

In the terrestrial data, the strongest correlations are found between genera and collections, and between formations and outcrop area (Supplementary Table 1), and these are also the only significant IT relationships. IT between genera and collections is strong and symmetric (Fig. 3g), whereas IT between formations and outcrop is weaker and approaches significant asymmetry in favour of outcrop (Outcrop>Formations; Fig. 5l)). In the conditional analyses, neither formations nor outcrop contain any significant information on genera or collections, while the latter show strong, symmetric CIT (Fig. 5g,h). Outcrop and formations show significant CIT, with CIT from outcrop to formations being stronger than in the opposite directions (Fig. 5i–k). Genera and collections show no CIT to outcrop or formations (Fig. 5l). The terrestrial CIT results thus reiterate the pairwise IT results, suggesting that terrestrial outcrop area contains a signal useful for predicting terrestrial formations, more than vice versa (Fig. 4).

### Palaeoenvironmental proxy data

Although correlations suggest significant relationships between marine formations and ^87/86^Sr, and between terrestrial formations and δ^18^O (Supplementary Table 1), we found no significant IT between the UK Phanerozoic records and global palaeoenvironmental proxy records, including sea level. These results should be regarded as tentative until regional proxy records (for example, regional flooding) are tested.

## Discussion

In combination, three of these findings, namely (i) strong symmetric IT, (ii) similar responses to outcrop area and (iii) cancelling out in conditional analyses, suggest that genera and collections are information redundant in the marine data. In the terrestrial data, only (i) can be demonstrated. The causal relationship between palaeodiversity and collections can in principle go both ways (Fig. 3), because palaeontologists’ collecting effort is to some extent guided by fossil richness in the field^19^,^33^,^34^,^40^. Therefore, caution is needed if collections are employed to ‘correct’ the palaeodiversity record. We did not assess occurrences (=localities) because of difficulties of definition and data compilation. However, we expect they would show the same patterns of potential RED as between collections and palaeodiversity.

Our results also suggest that marine formations can be considered information redundant with respect to palaeodiversity and collections. Although changes in fossil richness are not part of the formal criteria for defining formations, both may be confounded by changes in the primary depositional environment, and greater environmental/faunal turnover may enable a finer partitioning of formations. If formation boundaries were independent of fossil diversity, then changes in average richness from one formation to the next should not differ significantly from the changes obtained after randomly shuffling the formations over a fixed distribution of genera. Here, we use UK Triassic–Jurassic data^19^ to show that cross-formation changes in average generic richness are greater than would be expected under independence (see Methods for randomization test description; Fig. 6). The differences are less significant in the regional subsets, partly due to smaller sample sizes, and are generally less significant in the terrestrial than in the marine formation sets (Supplementary Figs 7–9). This covariation suggests that formations should not be considered a measure of rock quantity or sampling that is derived independently of changes in observed fossil diversity, and thus strengthens the argument of the RED. However, in line with the IT results, formations are less redundant with palaeodiversity in the terrestrial data than in the marine data.

Outcrop area, on the other hand, is a potential sampling proxy that may drive, but is not driven by, palaeontological research effort or fossil richness in the field. Our analysis suggests that outcrop area does contain a signal useful for predicting richness, collections and formations in the marine data (Fig. 4). Although this result may seem to favour the CC, it is not strictly a test of the CC hypothesis. Richness, collections, formations and outcrop area are all to some extent confounding factors, and thus bound by common-cause relationships in the general, statistical sense. However, the CC hypothesis as used in the palaeodiversity literature states more specifically that covariation between palaeodiversity and the amount of sedimentary rock is observed because both respond to similar environmental drivers, such as the degree of continental flooding^11^. To test this, we ideally need palaeoenvironmental proxies that are separate from the rock quantity proxy (for example, Hannisdal and Peters^13^). Outcrop area is a proxy that may reflect relative changes in the extent of continental flooding (primary depositional/shelf area), but also subsequent erosion and preservation (rocks available for sampling). Hence, in the absence of a regional flooding proxy, the outcrop area results are consistent with both RRB and CC hypotheses. Regardless of conditioning on outcrop area or formations, CIT between palaeodiversity and collections remains strong and symmetric, as expected under RED. If CC had acted to drive both richness and collections, but collections were the main cause of richness variation via anthropogenic sampling without RED, then we would expect asymmetric CIT from collections to palaeodiversity conditioned on outcrop area. If we assume that outcrop area represents CC mechanisms more than RRB, then our finding that CIT from outcrop to palaeodiversity is stronger than CIT from formations to palaeodiversity would suggest CC. However, formations seem to provide very little information not already contained in the other variables, and irrespective of whether CC or RRB is acting, the bidirectional IT between formations and palaeodiversity, together with the formation randomization results, are more simply explained by RED. In the marine realm, a species-area effect could result from either a continental flooding effect, in line with CC, or a sampling effect, in line with RRB; there is greater spatiotemporal continuity of deposition, facies tend to intergrade, and formations may be indirectly influenced by changes in fossil diversity. Marine formations may therefore be more redundant with palaeodiversity, and changes in area/volume may be more important than changes in habitat-specific preservation potential, thus outcrop better predicts diversity. In the terrestrial realm, there is no obvious area effect, little spatiotemporal continuity of deposition, and fossil preservation is more linked to habitat.

Sedimentary processes may also account for differences between the marine and terrestrial realms, with consistent good preservation in marine deposits and sporadic preservation in terrestrial sediments^19^,^41^. Therefore, the amount of rock preserved and accessible to palaeontologists may partly control palaeodiversity in the marine realm, whilst any species-area effect (whether RRB or CC driven) is overwhelmed by sporadic preservation in the terrestrial realm^19^. It could also be that we see a CC mechanism operating in the marine realm^11^,^13^,^42^, but not in the terrestrial realm^43^ given the lack of hypothesized common drivers of sedimentation and diversity in the terrestrial realm. Either way, outcrop area is a generalized measure of rock availability and the degree of exposed bedrock can vary significantly across rocks at different altitudes, locations, and ages^36^,^44^. In any case, a singular sampling metric will fail to capture all bias affecting palaeodiversity^5^,^20^,^33^, and unless CC can be ruled out, outcrop area should not be used as a simple correction factor.

It would be ideal if a sampling proxy could be found that would allow palaeontologists to remove bias from their empirical palaeodiversity data. Several approaches to correcting the fossil record have been proposed, but our results suggest caution in applying these. Our results do not address the rarefaction and SQS sampling correction approaches adopted by Alroy^1^,^25^, but they do have a strong bearing on methods that use residuals from comparisons of fossil record (palaeodiversity) and rock record (collection counts, formation counts, map areas) time series.

First, we argue that it is wrong to claim that outcrop areas calculated from geological maps are a meaningful measure of sampling^5^,^7^,^17^,^27^ because we find limited evidence that outcrop areas equate to rock accessibility^20^,^33^,^34^,^36^,^44^. Of course, outcrop areas may include some aspect of sampling, but this is probably overwhelmed by a number of unpredictable factors, such as (1) outcrop areas incorporate tracts of unpredictable size that are devoid of fossiliferous rocks^29^, (2) they include other tracts of unpredictable size that are concealed beneath soil^29^,^36^,^44^, (3) these first two factors are heavily dependent on rock facies, so outcrop areas of different ages cannot be compared unless they comprise similar facies distributions^19^,^33^,^35^,^41^, (4) fossil recovery depends on the maturity of sampling of individual localities, so equivalent outcrop areas of different ages or in different parts of the world may be sampled to a greater or lesser extent and so cannot be compared as metrics of sampling and (5) the species-area effect and its relationship with flooding may not be (log-) linear or even monotonic^45^, and outcrop area is unlikely to be related to species counts in a simple way. However, this problem is generally avoided as most studies do not assume a linear relationship between outcrop area and species counts. As outcrop area represents the total amount of rock preserved more effectively than it represents the amount of accessible rock (that is, exposed bedrock), the IT between outcrop area and palaeodiversity may indicate a stronger role for CC relative to RRB. However, independent, regional environmental proxy records are required to further resolve this.

Further, our results from correlation, IT and formation randomization tests all indicate that formation and collection counts may be influenced by fossil diversity, and so neither can be used as an independent sampling proxy to correct the palaeodiversity record, as formerly recommended by some^8^,^14^,^15^. Proceeding to use such methods to generate a ‘bias-corrected’ palaeodiversity curve risks obliterating true biological signal. The question is probably much more complex than has often been assumed. Ultimately, palaeodiversity reconstruction will have to address a multitude of biasing factors, by defining a space of hypotheses in the form of stochastic models^46^, and confronting these models with available sources of palaeontological, stratigraphic and geochemical data, using formal inversion methods that more realistically account for the time-varying uncertainty in both models and data.

## Methods

### Data

**Figure 1:**
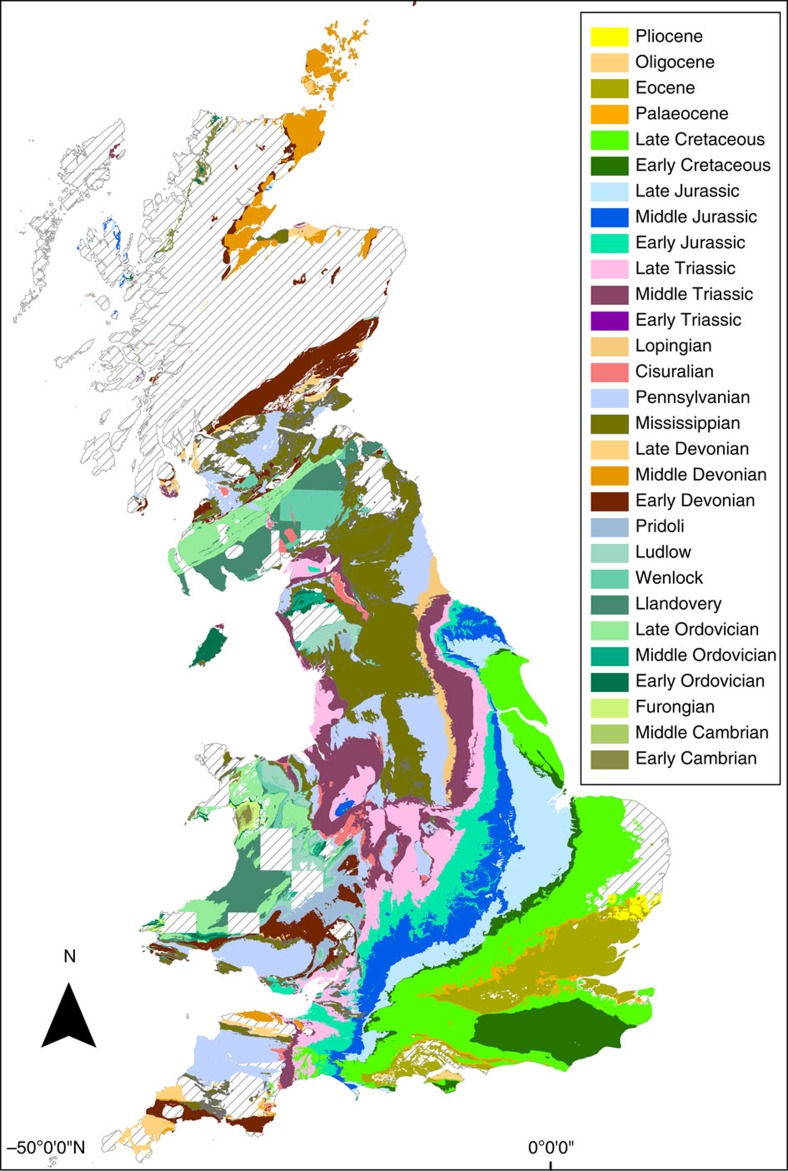
The Phanerozoic record of Great Britain. Geological map of British Phanerozoic sedimentary rock outcrop at the Epoch level (British Geological Survey DiGMapgb-50 1:50,000). Hashed areas represent extent of igneous and metamorphic rocks or areas that have not been digitally mapped. There are no data recorded from the Guadalupian or Miocene.

**Figure 3:**
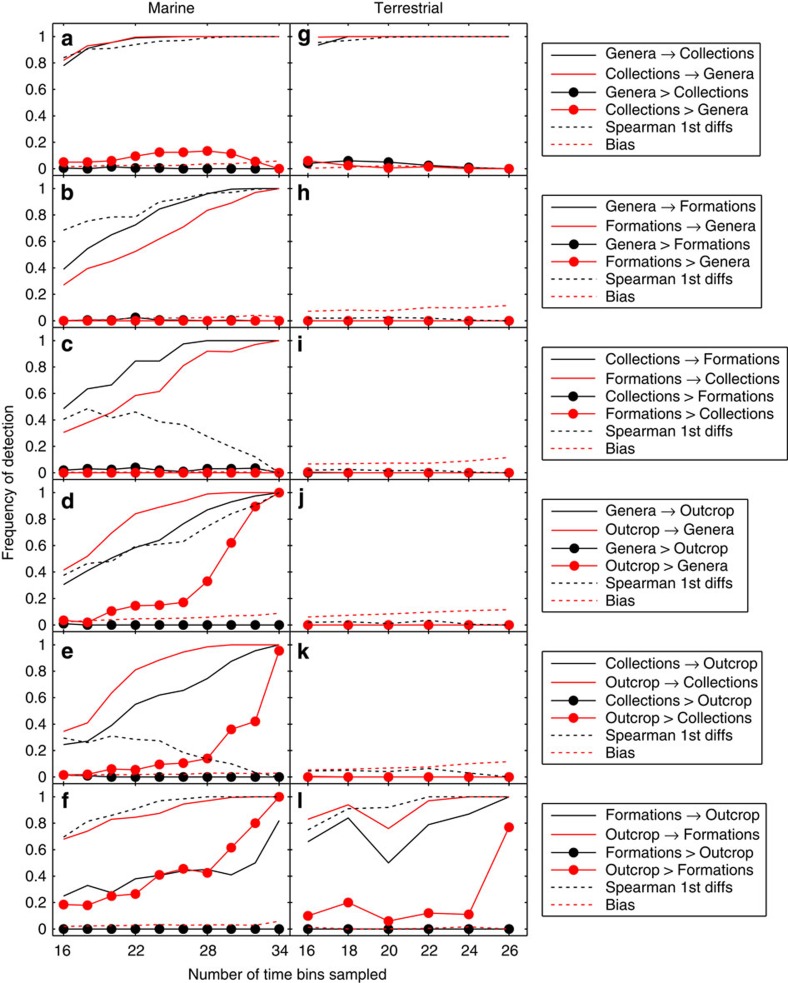
Directional IT and correlations between sampling proxies and palaeodiversity in the marine and terrestrial data. Frequency of detection represents the proportion of significant results (alpha=0.01) in 500 analyses by repeated random sampling of the original time series. Detection frequency (statistical ‘sensitivity’) is evaluated as a function of the number of time bins (epochs) sampled in each iteration. For each pair of variables X and Y (indicated in the plot legend for each row of panels), solid lines correspond to significant IT in each direction (X→Y and Y→X). Filled circles correspond to a detected difference in magnitude between the two (where X>Y denotes that X→Y is significantly greater than Y→X), which represents significant asymmetry or directionality in the IT. Stippled black line corresponds to significant Spearman rank-order correlations on first differences. Stippled red line represents an index on [0,1] of potential bias due to differences in non-stationarity, where bias values of 0.1 or less are considered negligible. See text for description of the results in each panel.

**Figure 4:**
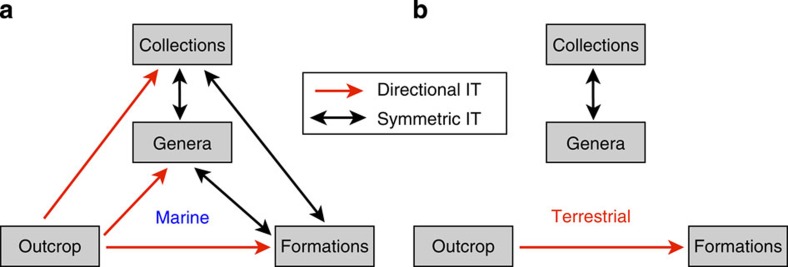
IT in the marine and terrestrial realms. Flowcharts summarizing IT results in **a**, marine data and **b**, terrestrial data, indicating symmetric (black arrows) and directional (red arrows) information flow.

**Figure 5:**
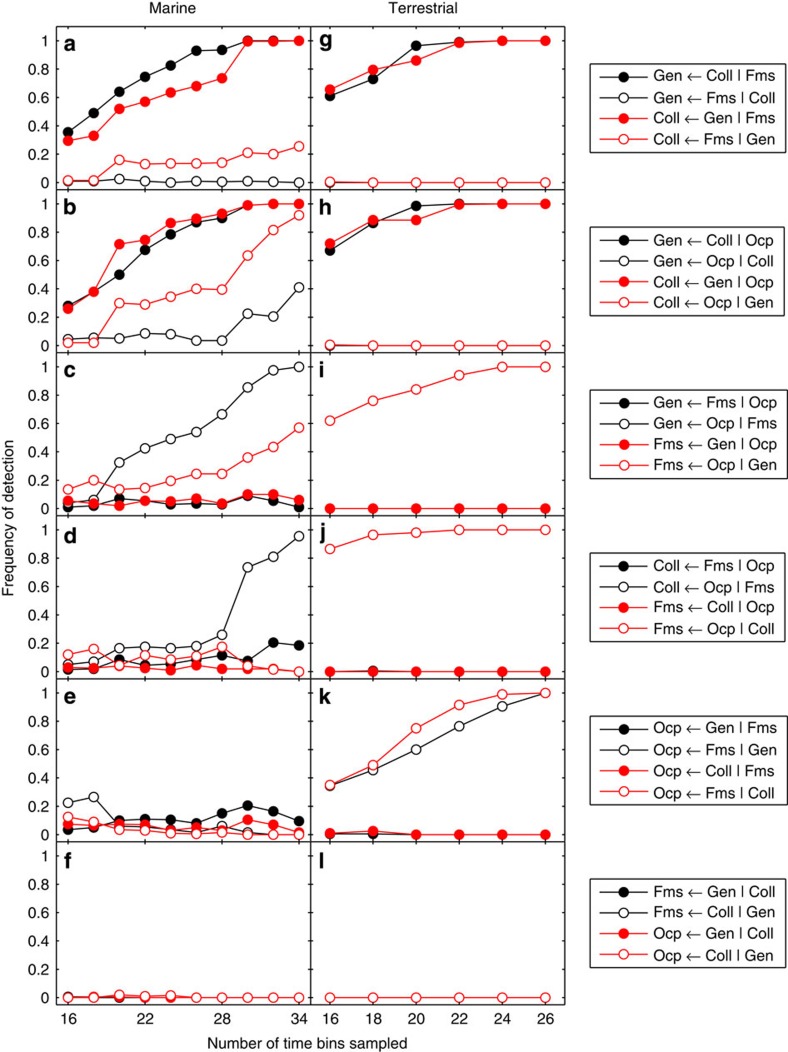
CIT amongst sampling proxies and palaeodiversity in the marine and terrestrial data. Frequency of detection represents the proportion of significant results (alpha=0.01) in 500 analyses by repeated random sampling of the original time series. Detection frequency (statistical ‘sensitivity’) is evaluated as a function of the number of time steps sampled in each iteration. For each set of three variables X, Y and Z (indicated in the plot legend for each row of panels), significant X←Y|Z implies that Y contains information useful for predicting changes in X beyond their mutual interaction with Z. Black and red filled circles correspond to switching the ‘response’ (X) and ‘driver’ (Y) variables for a fixed conditioning variable Z (that is, opposite directions X←Y|Z and Y←X|Z). For a given colour, filled and open circles correspond to switching the ‘driver’ and conditioning variable for a fixed ‘response’ variable. See text for description of the results in each panel. Gen, Genera; Coll, Collections; Fms, Formations; Ocp, Outcrop.

**Figure 6:**
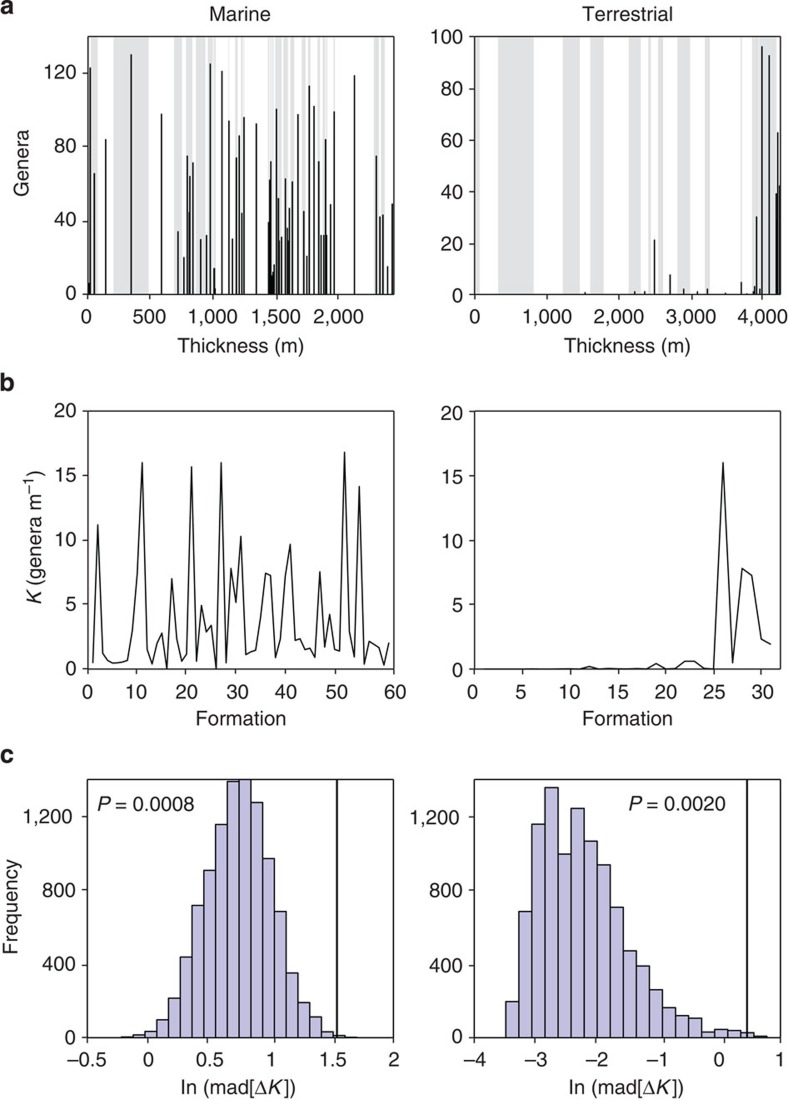
Testing for fossil formation independence in Triassic–Jurassic rocks. (**a**) Cumulative thickness of stacked formations (alternating white/grey-shaded), with the number of reported genera (black bars) indicated at the centre of each formation. (**b**) Average fossil richness *K* (genera per m) in each formation. (**c**) Comparing the observed volatility of *K* (black line), measured as the mean absolute deviation (mad) of first differences (Δ*K*), against null distributions for 10,000 shuffles of *K* (randomly reordering the formation stack while keeping the distribution of genera fixed). *P*-values for the permutation test represent the proportion of values in the null distribution (histogram) that exceed the observed volatility.

Generic occurrence data, formation counts and collection counts were obtained from the PaleoDB ( http://paleobiodb.org/) in January 2013. PaleoDB data was derived from 31,321 occurrences of 4,029 genera within 2,829 collections. As a whole, the PaleoDB varies in completeness through geological time. Therefore, it is inevitable that this British subset should suffer from this same issue. In particular, the time bins of the Late Carboniferous, the Permian and the Miocene appear particularly under-sampled, whereas the Lower Palaeozoic, Jurassic, Cretaceous and Eocene appear to be the most thoroughly sampled. However, as this study is primarily aimed at detecting sampling issues, this should not cause a problem regarding the interpretation of our results. Outcrop area measurements were obtained from the British Geological Survey digital bedrock geology DiGMapgb-50 of the UK (1:50,000; Fig. 1). Palaeoenvironmental time series were resampled from the original data sources (Hannisdal and Peters^13^) and bin-averaged in the time bins of the UK rock and fossil data.

Thickness and palaeodiversity for UK Triassic–Jurassic formations were obtained from Dunhill *et al.*^19^ for (i) sequence across the entire UK, (ii) the Wessex basin, (iii) the East Midlands basin and (iv) the Yorkshire basin. Formations were stacked in stratigraphic sequence (using mean thickness across different locations for each formation in the total sequence) with the number of fossil genera *G*_*i*_ assigned to a single point in the centre of the *i*th formation. Average fossil richness *K*_*i*_=*G*_*i*_/*Z*_*i*_ (genera per m) was calculated for each formation *i* with thickness *Z*_*i*_. Significance tests were carried out on the mean absolute deviation of first differences in the observed *K* against 10,000 shuffles of *K* calculated by keeping *G* fixed but randomly reordering *Z*, to test whether variation across formation boundaries is greater than would be expected by chance.

### Statistical analysis

Spearman rank-order correlations were calculated on first differences. IT was calculated pairwise, using 500 amplitude-adjusted Fourier transform surrogates to establish significance of IT in each direction, X→Y, and Y→X. If one or both, then the difference between IT in opposite directions was compared to that of the surrogate distribution to test for significantly asymmetric (directional) information flow (X>Y or Y>X, where X>Y denotes that X→Y is significantly greater than Y→X). CIT was calculated on sets of three variables to test if the IT between X and Y was still significant when taking into account their common interaction with a third variable, Z^38^. To evaluate whether or not differences in non-stationarity could bias the IT between two time series, a bias index was calculated from the KPSS test^47^, such that a maximum value of one indicates different non-stationarity at all time lags, and a minimum value of zero indicates no differences. If needed to minimize bias, time series were detrended (linearly, or using a higher-order polynomial fit) and power transformed to stabilize the variance (Box–Cox). The Eocene was removed to avoid excessive data regularization prompted by the Eocene diversity ‘spike’. All records were normalized to mean zero and unit standard deviation before analysis. Correlation/IT results are presented as sensitivity analyses, giving the proportion of significant findings (frequency of detection) in 500 analyses by iterative sampling of the original data, plotted as a function of the number of time bins sampled in each iteration (see Supplementary Methods for more details).

## Author contributions

A.M.D. compiled the palaeodiversity data and compiled and analysed the spatial sampling proxy data using ArcGIS v10.1. B.H. performed the information transfer analysis and formation randomization tests. A.M.D., B.H. and M.J.B. designed the study and wrote the paper.

## Additional information

**How to cite this article**: Dunhill, A. M. *et al.* Disentangling rock record bias and common-cause from redundancy in the British fossil record. *Nat. Commun.* 5:4818 doi: 10.1038/ncomms5818 (2014).

## Supplementary Material

Supplementary Figures, Table, Methods and References Supplementary Figures 1-9, Supplementary Table 1, Supplementary Methods and Supplementary References

Supplementary Data 1 Raw times series and formation boundary data.

## Figures and Tables

**Figure 2:**
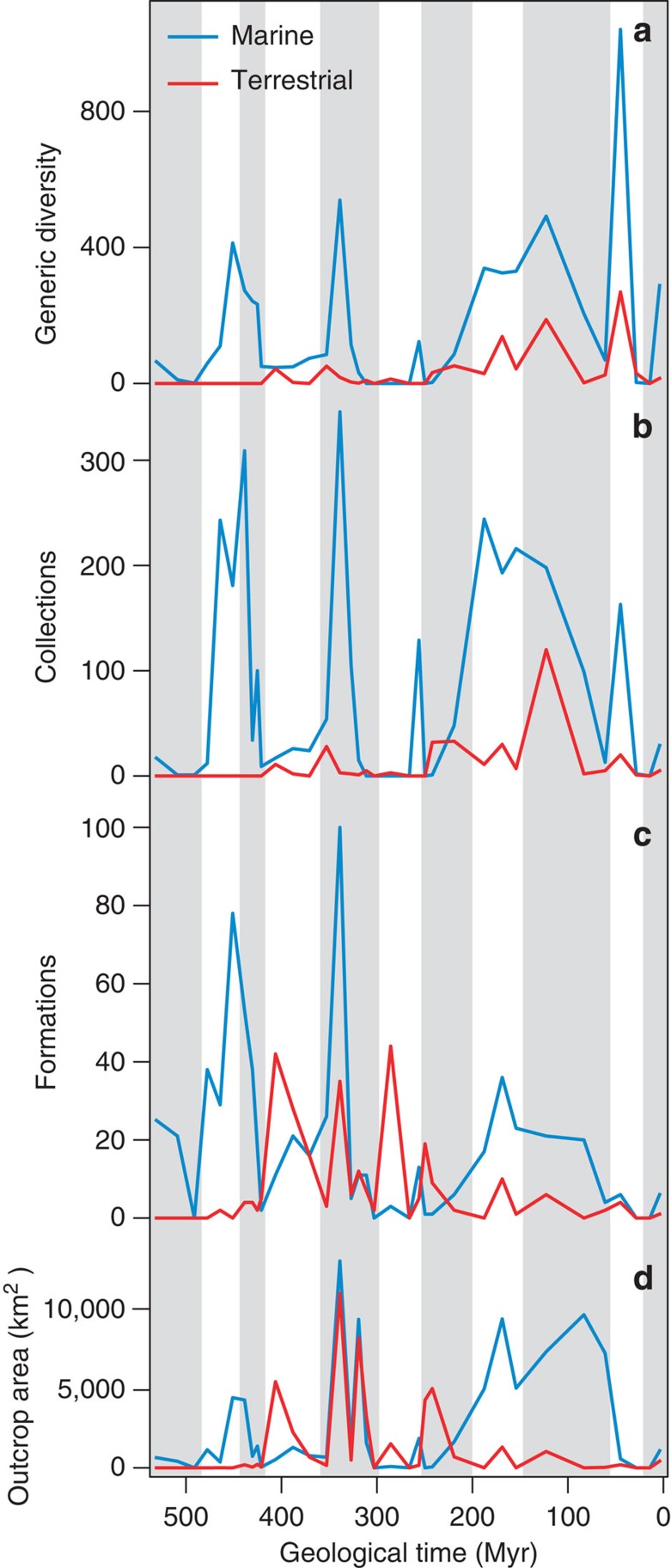
Palaeodiversity and sampling proxies. Time series of (**a**) generic palaeodiversity, (**b**) collections, (**c**) formations and (**d**) outcrop area through the British marine and terrestrial Phanerozoic at Epoch level.
