# Supplementary material for: Disentangling rock record bias and common-cause from redundancy in the British fossil record
Source: Nat Commun. 2014 Sep 4;5:4818. doi: 10.1038/ncomms5818 (PMC4164781; doi:10.1038/ncomms5818)
Supplement: Supplementary Figures, Table, Methods and References — Supplementary Figures 1-9, Supplementary Table 1, Supplementary Methods and Supplementary References [file ncomms5818-s1.pdf]

Supplementary Information

Disentangling rock record bias and common-cause from redundancy in the British fossil record

A.M. Dunhill, B. Hannisdal, M.J. Benton

1. Supplementary Figures

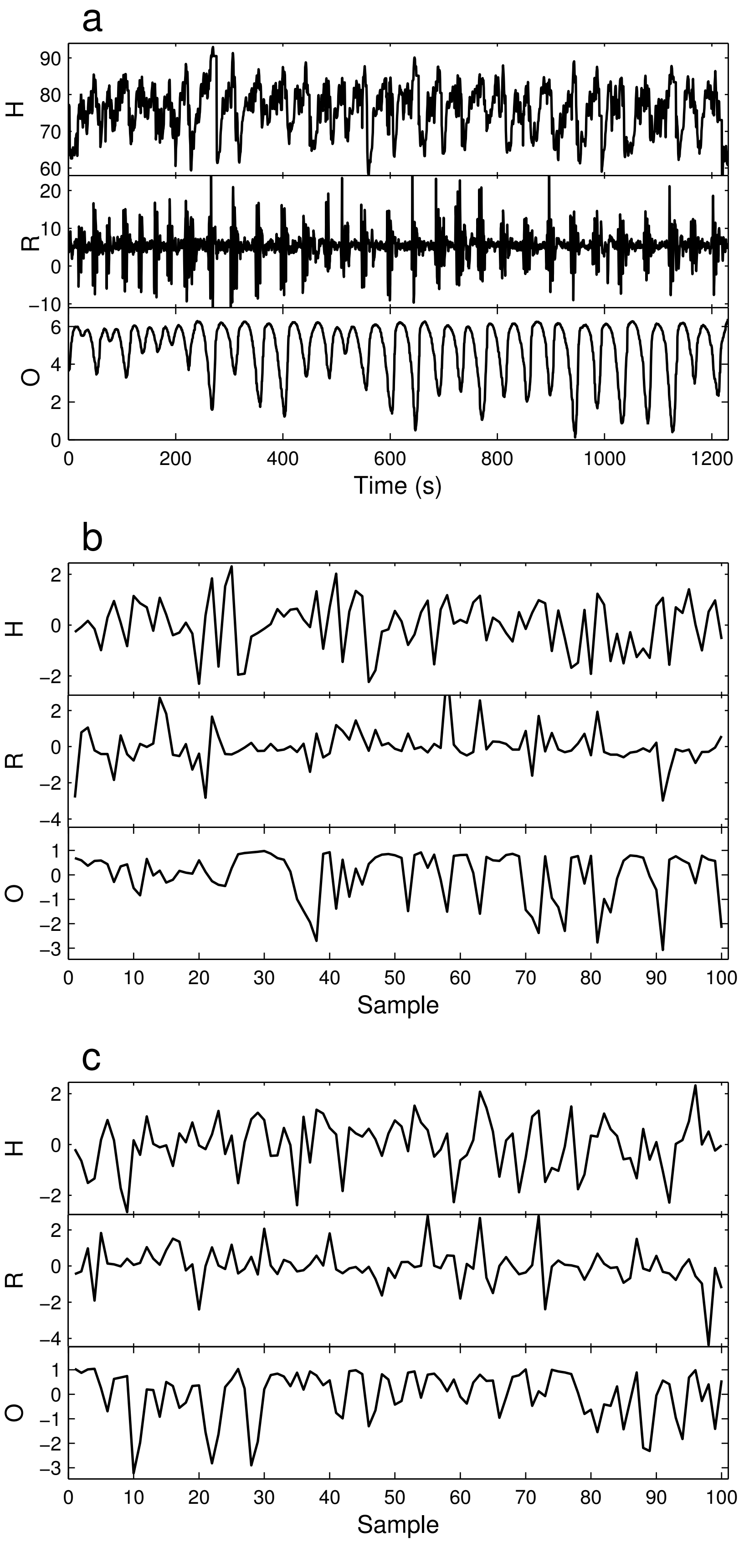

Supplementary Figure 1 | Sleep apnea. (**a**) Monitored heart rate (H), respiration (R), and blood oxygen level (O) in a sleep apnea patient[1]. (**b**) The same data sampled at 100 randomly selected time steps. (**c**) AAFT surrogate realization of the data in **b**.

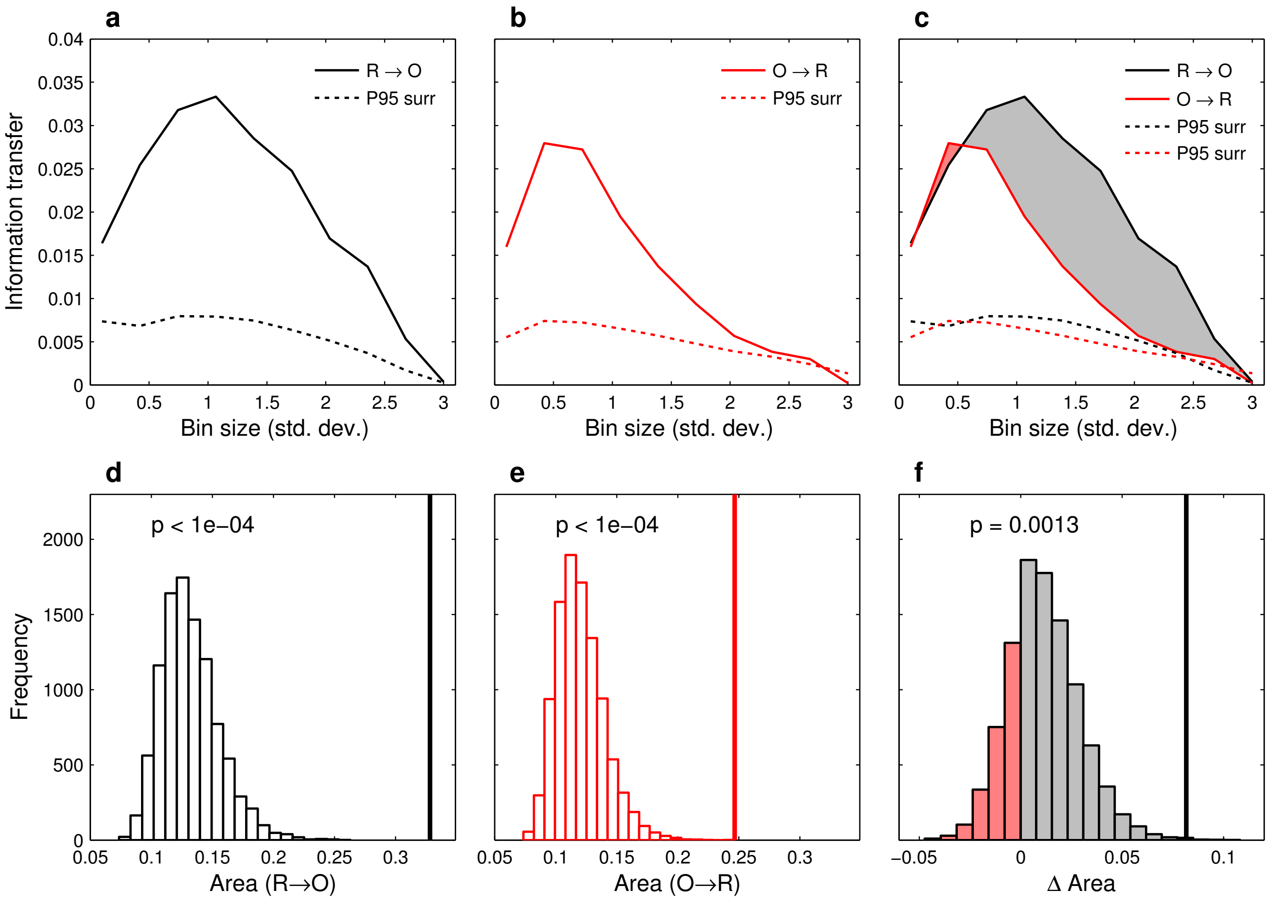

Supplementary Figure 2 | An example of the three significance tests involved in the directional IT analysis between two time series. This analysis is performed on the R and O records in Supplementary Fig. 1b (i.e. 100 random samples of the original time series). Significance is established using 10,000 pairs of surrogate time series (e.g. Supplementary Fig. 1c). (**a**) IT from R to O (R→O) as a function of the bin size used for gridding the data (in units of standard deviation for normalized data). Stippled line represents the 95th percentile of the surrogate IT distribution. All IT values are here plotted relative to the surrogate median value. (**b**) Same as **a**, but in the opposite direction (O→R). (**c**) Superimposing the IT curves in both directions to highlight the difference in the area under the two curves (gray and red shading represent opposite sign). (**d**) Using the area under the IT curve in panel **a** as an informal measure of total IT (thus ignoring possible scale dependence), we see that the IT from R to O (vertical line) is significantly greater than that of 10,000 surrogate pairs (histogram). (**e**) Same as **d**, but in the opposite direction (area under the curve in panel **b**). (**f**) If one or both of the tests in **d** and **e** are significant, then we test whether the asymmetry (vertical line; corresponding to the difference between the gray and the red shaded areas in panel **c**) is greater than that of the surrogates (histogram; shading indicating which area is larger), suggesting a significant asymmetry of information flow (denoted R > O).

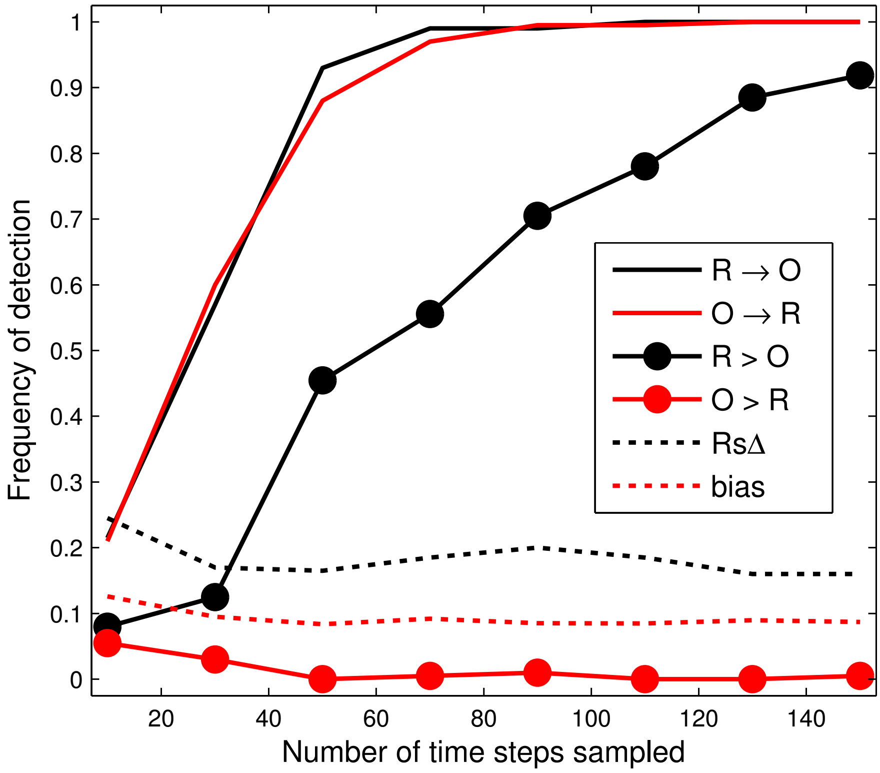

Supplementary Figure 3 | Evaluating the robustness of IT between R and O. Values are the frequency of significant results (500 iterations, alpha = 0.05) as a function of the number of time steps sampled. Solid lines correspond to significant IT in each direction (R→O and O→R), filled circles correspond to significant *asymmetry* between the two (R>O or O>R). Stippled black line corresponds to significant Spearman rank-order correlations on first differences (RsΔ). Stippled red line represents an index of potential bias due to differences in non-stationarity, which can be plotted on the same scale (1 being maximum bias). This low level of bias is unlikely to explain the observed IT (see Supplementary Fig. 5).

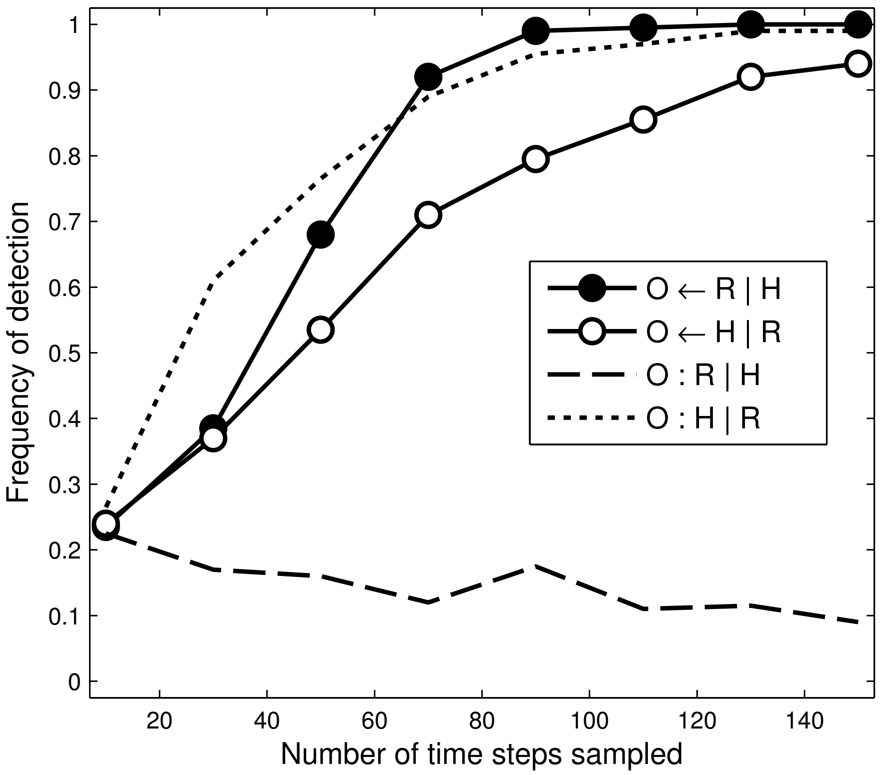

Supplementary Figure 4 | Conditional IT sensitivity analysis. Conditional IT from R to O is clearly significant when taking into account their common interaction with H (O ← R | H). The conditional IT from H to O given R (O ← H | R) is weaker, but also approaches significance. In contrast, the partial Spearman correlation on first differences between O and H given R (O : H | R) is significant, whilst the partial correlation between O and R given H is not.

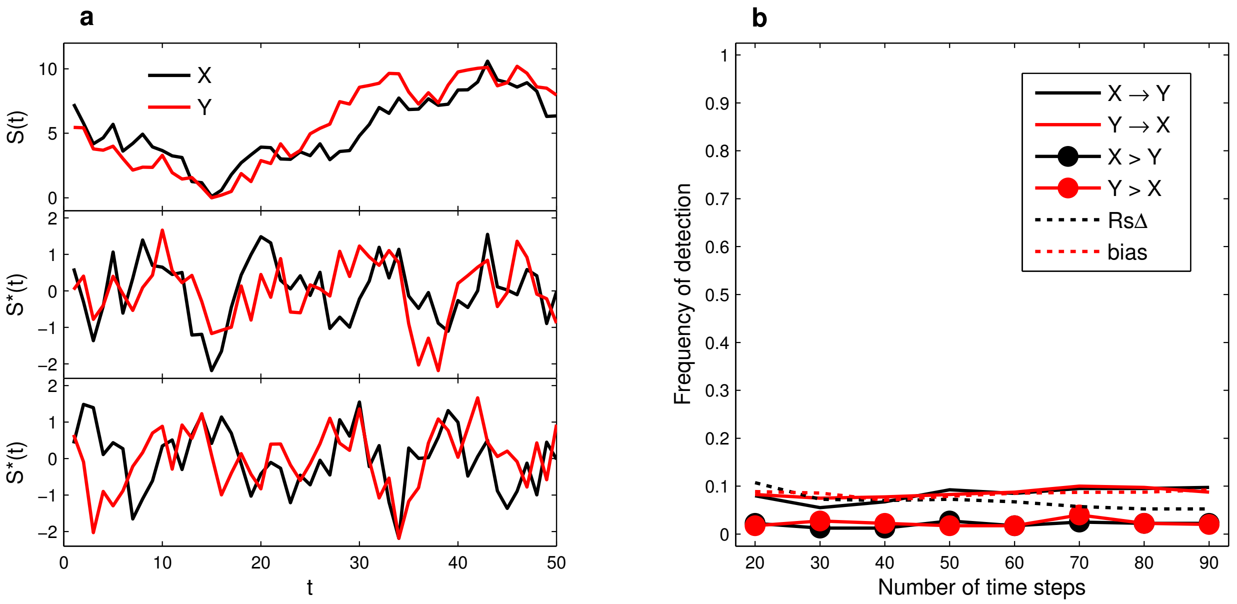

Supplementary Figure 5 | Data pre-processing. (**a**) Upper panel: a pair of unbiased random walks with a length of 50 time steps. Middle panel: the same time series after detrending by subtracting a best-fit 5th-order polynomial, and normalization. Lower panel: AAFT surrogates of the two pre-processed time series. (**b**) IT sensitivity analysis (cf. Supplementary Fig. 3) on pairs of random walks after pre-processing. Values represent the frequency of significant IT (i.e. false detection rate at alpha = 0.05) for random walks of different length.

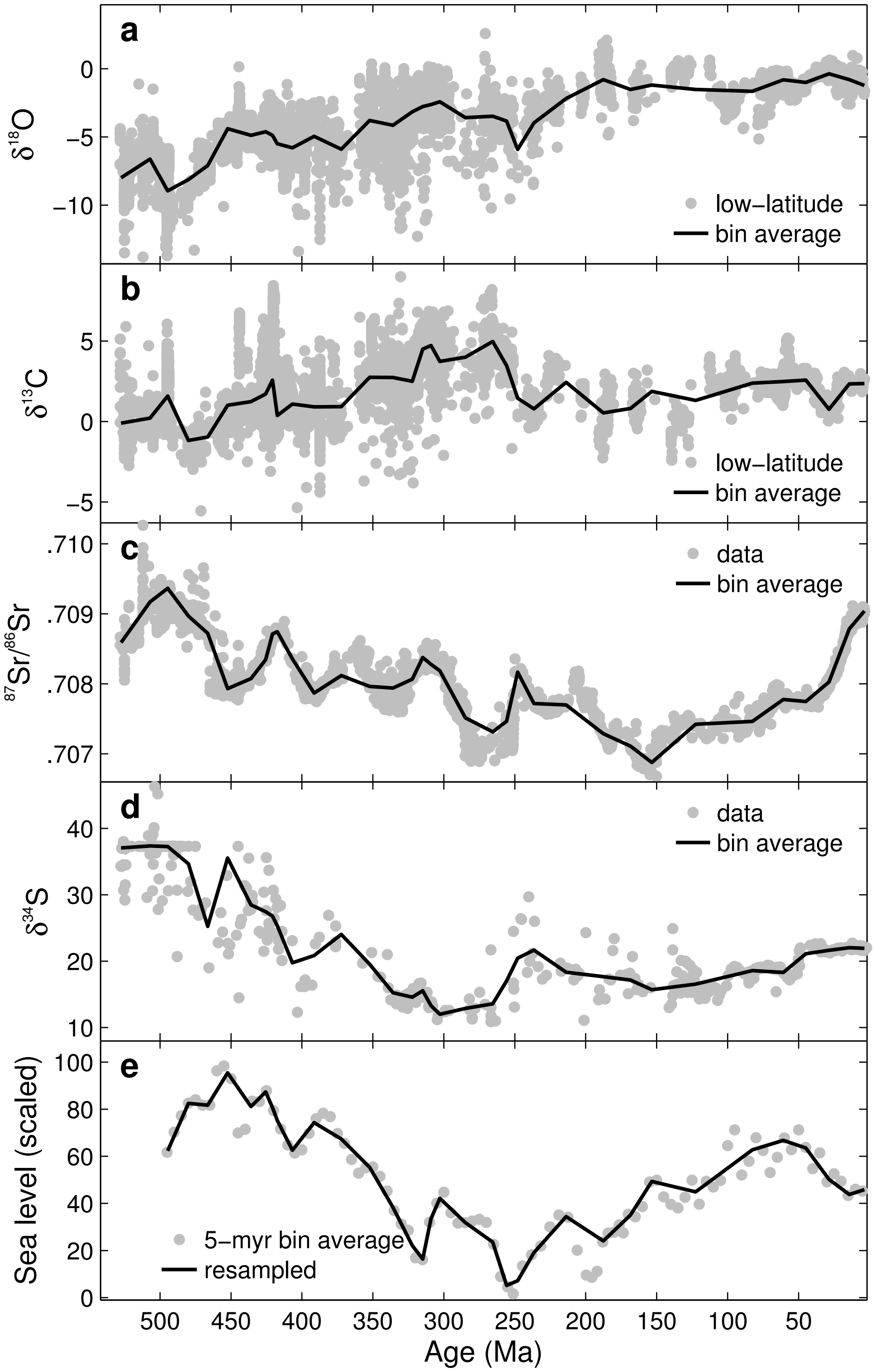

Supplementary Figure 6 | Environmental proxies. Isotope ratios from marine carbonates[2]: (**a**) $\delta^{18}O$, (**b**) $\delta^{13}C$, (**c**) $\delta^{34}S$, and (**d**) $^{87}Sr/^{86}Sr$. (**e**) Global estimate of continental flooding[3]. Black lines represent bin-averages using the time bins of the UK Phanerozoic records.

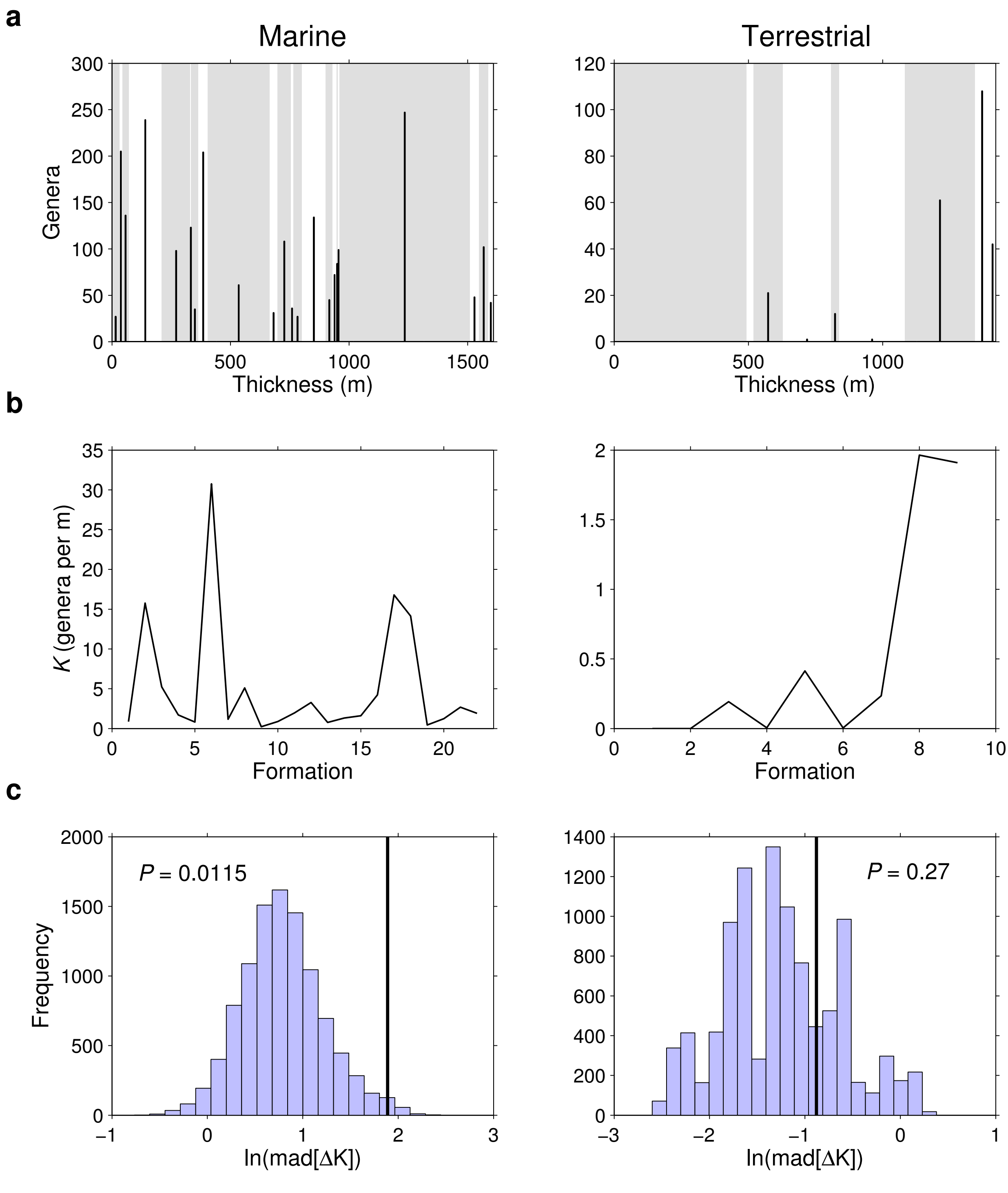

Supplementary Figure 7 | Testing for fossil-formation independence in Triassic-Jurassic rocks of the Wessex basin. (**a**) Cumulative thickness of stacked formations (alternating white/grey-shaded), with the number of reported genera (black bars) indicated at the centre of each formation. (**b**) Average fossil richness K (genera per m) in each formation. (**c**) Comparing the observed volatility of K (black line), measured as the mean absolute deviation (mad) of first differences (ΔK), against null distributions for 10,000 shuffles of K (randomly reordering the formation stack while keeping the distribution of genera fixed). P-values for the permutation test represent the proportion of values in the null distribution (histogram) that exceed the observed volatility.

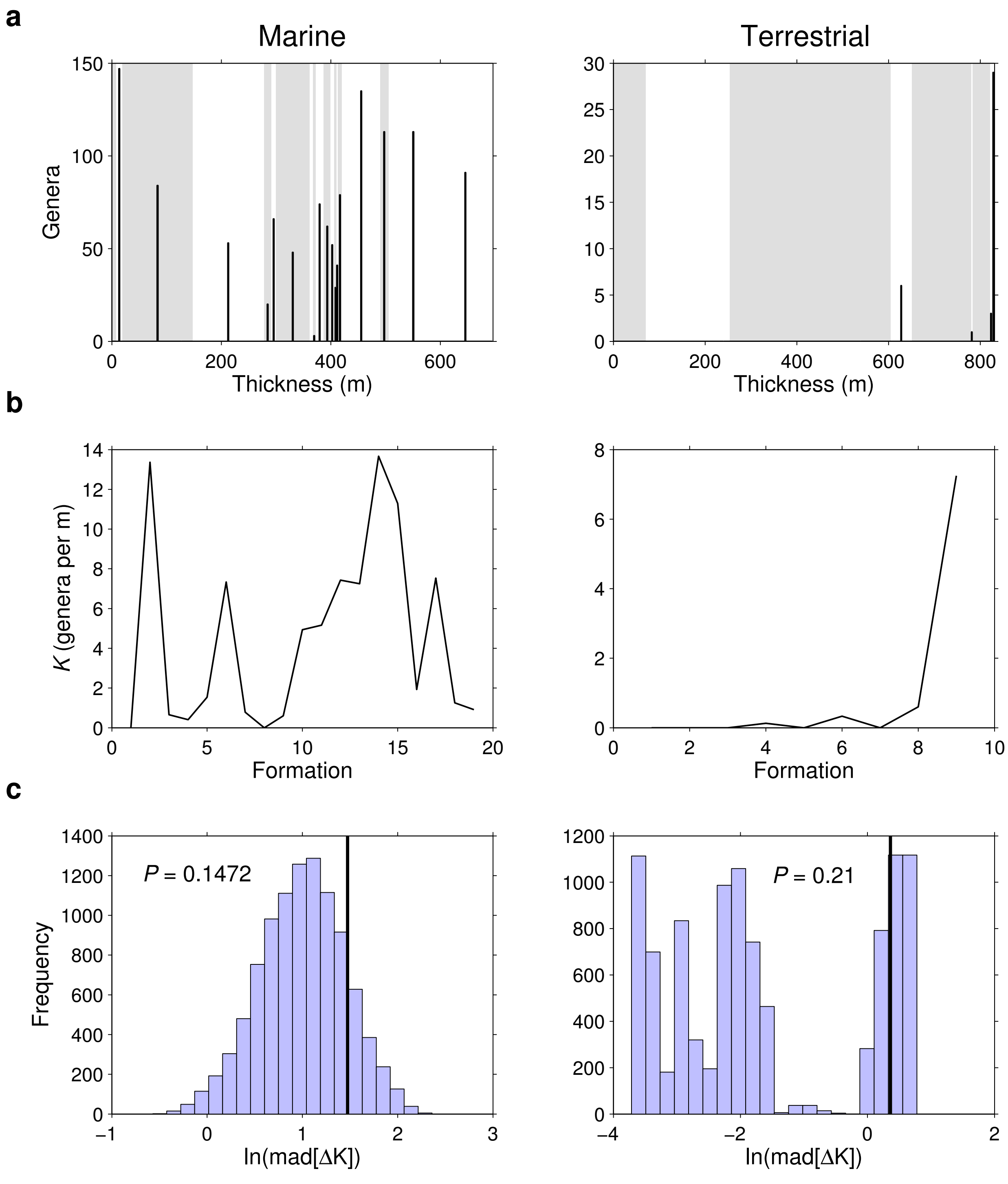

Supplementary Figure 8 | Testing for fossil-formation independence in Triassic-Jurassic rocks of the East Midlands basin. (**a**) Cumulative thickness of stacked formations (alternating white/grey-shaded), with the number of reported genera (black bars) indicated at the centre of each formation. (**b**) Average fossil richness K (genera per m) in each formation. (**c**) Comparing the observed volatility of *K* (black line), measured as the mean absolute deviation (mad) of first differences (ΔK), against null distributions for 10,000 shuffles of *K* (randomly reordering the formation stack while keeping the distribution of genera fixed). *P*-values for the permutation test represent the proportion of values in the null distribution (histogram) that exceed the observed volatility.

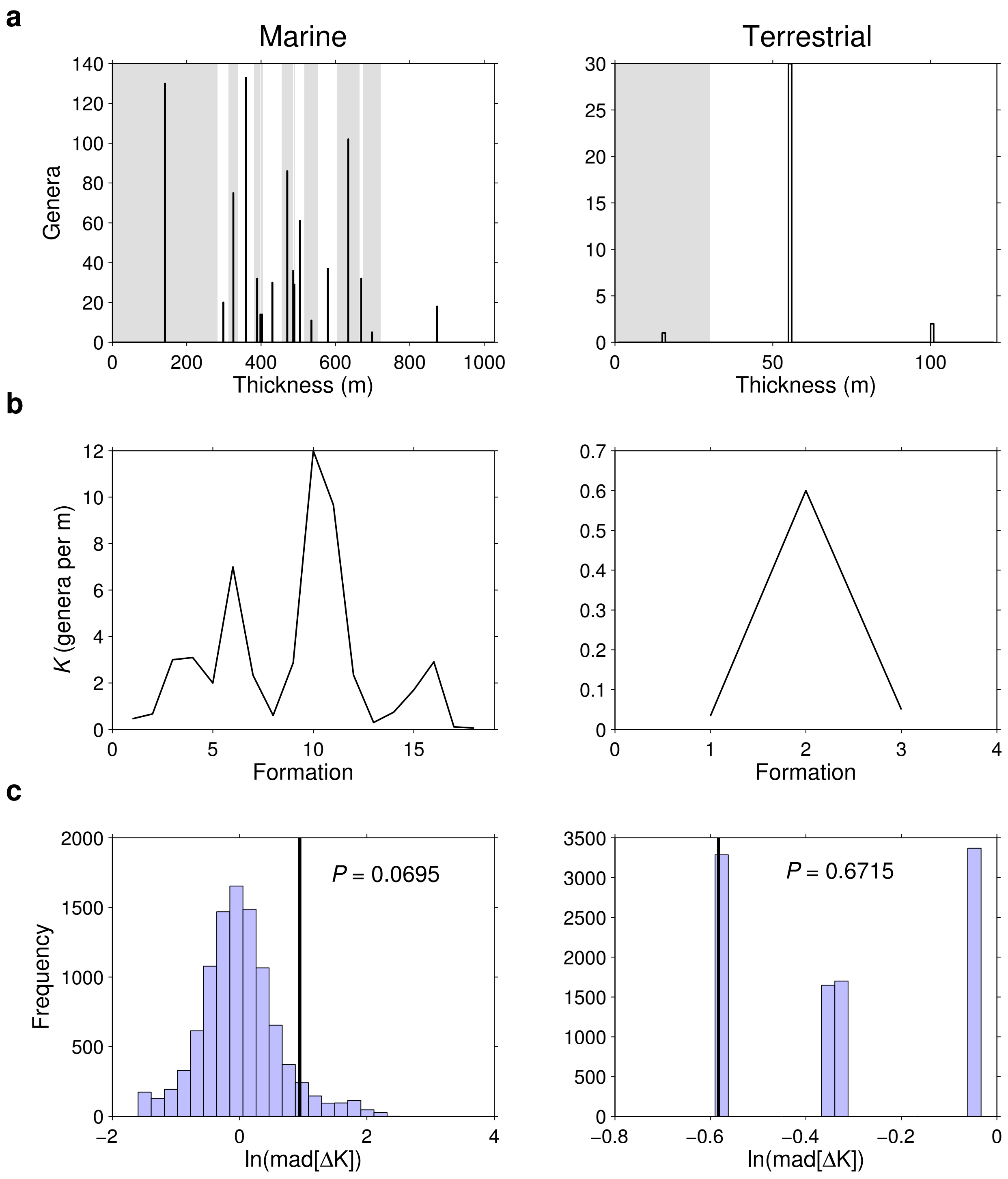

Supplementary Figure 9 | Testing for fossil-formation independence in Triassic-Jurassic rocks of the Yorkshire basin. (**a**) Cumulative thickness of stacked formations (alternating white/grey-shaded), with the number of reported genera (black bars) indicated at the centre of each formation. (**b**) Average fossil richness K (genera per m) in each formation. (**c**) Comparing the observed volatility of K (black line), measured as the mean absolute deviation (mad) of first differences (ΔK), against null distributions for 10,000 shuffles of K (randomly reordering the formation stack while keeping the distribution of genera fixed). P-values for the permutation test represent the proportion of values in the null distribution (histogram) that exceed the observed volatility.

2. Supplementary Tables

Supplementary Table 1| Spearman rank correlation tests between sampling proxies, environmental proxies, and palaeodiversity on first differenced (RsΔ) data for marine and terrestrial data sets. * significant at $p < 0.05$, ** significant after false discovery rate correction using the method of Benjamini and Hochberg[4].

	Marine	Terrestrial
Genera ~ collections	RsΔ = 0.68, $p < 0.001$**	RsΔ = 0.93, $p < 0.001$**
Genera ~ formations	RsΔ = 0.66, $p < 0.001$**	RsΔ = 0.43, $p = 0.03$*
Genera ~ outcrop	RsΔ = 0.41, $p = 0.02$*	RsΔ = 0.41, $p = 0.04$*
Collections ~ formations	RsΔ = 0.38, $p = 0.03$*	RsΔ = 0.35, $p = 0.09$
Collections ~ outcrop	RsΔ = 0.25, $p = 0.15$	RsΔ = 0.39, $p = 0.05$*
Formations ~ outcrop	RsΔ = 0.61, $p < 0.001$**	RsΔ = 0.78, $p < 0.001$**
Genera ~ $\delta^{18}O$	RsΔ = 0.02, $p = 0.89$	RsΔ = -0.06, $p = 0.78$
Genera ~ $\delta^{13}C$	RsΔ = 0.02, $p = 0.9$	RsΔ = 0.16, $p = 0.43$
Genera ~ $^{87}Sr/^{86}Sr$	RsΔ = 0.35, $p = 0.04$*	RsΔ = 0.12, $p = 0.55$
Genera ~ $\delta^{34}S$	RsΔ = 0.07, $p = 0.69$	RsΔ = -0.15, $p = 0.48$
Genera ~ sea level	RsΔ = -0.03, $p = 0.88$	RsΔ = -0.04, $p = 0.86$
Collections ~ $\delta^{18}O$	RsΔ = -0.05, $p = 0.78$	RsΔ = -0.09, $p = 0.68$
Collections ~ $\delta^{13}C$	RsΔ = 0.02, $p = 0.9$	RsΔ = 0.1, $p = 0.64$
Collections ~ $^{87}Sr/^{86}Sr$	RsΔ = 0.3, $p = 0.08$	RsΔ = 0.05, $p = 0.82$
Collections ~ $\delta^{34}S$	RsΔ = -0.34, $p = 0.05$*	RsΔ = 0.002, $p = 0.99$
Collections ~ sea level	RsΔ = -0.19, $p = 0.29$	RsΔ = -0.09, $p = 0.68$
Formations ~ $\delta^{18}O$	RsΔ = 0.14, $p = 0.43$	RsΔ = -0.66, $p < 0.001$**
Formations ~ $\delta^{13}C$	RsΔ = 0.09, $p = 0.59$	RsΔ = -0.13, $p = 0.53$
Formations ~ $^{87}Sr/^{86}Sr$	RsΔ = 0.49, $p = 0.004$**	RsΔ = -0.11, $p = 0.61$
Formations ~ $\delta^{34}S$	RsΔ = -0.04, $p = 0.82$	RsΔ = 0.06, $p = 0.79$
Formations ~ sea level	RsΔ = 0.03, $p = 0.89$	RsΔ = -0.25, $p = 0.22$
Outcrop ~ $\delta^{18}O$	RsΔ = 0.01, $p = 0.95$	RsΔ = -0.5, $p = 0.01$*
Outcrop ~ $\delta^{13}C$	RsΔ = 0.16, $p = 0.38$	RsΔ = -0.08, $p = 0.7$
Outcrop ~ $^{87}Sr/^{86}Sr$	RsΔ = 0.19, $p = 0.28$	RsΔ = -0.07, $p = 0.73$
Outcrop ~ $\delta^{34}S$	RsΔ = 0.06, $p = 0.74$	RsΔ = 0.13, $p = 0.53$
Outcrop ~ sea level	RsΔ = -0.05, $p = 0.78$	RsΔ = -0.3, $p = 0.14$

3. Supplementary Methods

The IT approach is described in more detail in Schreiber[5], Verdes[6], and Hannisdal[7,8]. Here we illustrate the IT analysis and its interpretation with a commonly used example from human physiology (Supplementary Fig. 1). Normally, breathing causes variations in the heart rate: when we inhale, the heart rate begins to increase, and when we exhale, it decreases. In addition, the heart rate responds to the partial pressure of oxygen in the arteries. However, the data in Supplementary Fig. 1a come from a patient suffering from sleep apnea[1], where breathing is halted during sleep, causing blood oxygen levels to fall, which eventually alerts the brain to resume breathing. Sleep apnea may thus disturb the usual patterns of interaction and feedback among the heart rate (H), respiration (R), and blood oxygen concentration (O). Intuitively, the anatomically obstructed breathing would be considered an important driver of the system in this case, but one that also responds to the oxygen "alarm", and the relationship between R and O will be highlighted here.

All three time series are sub-sampled at randomly spaced time steps (e.g. Supplementary Fig. 1b), in analogy to sparse geological records with only relative age control. In line with common practice, we report simple correlations between time series in the form of Spearman rank-order correlation after aggressive detrending by first differencing. The IT, on the other hand, involves binning the observed amplitudes into histograms of the distribution of possible state transitions, and does *not* use differencing. Certain precautions therefore have to be made when testing for significance, including data pre-processing (see below), and the use of amplitude-adjusted Fourier transform (AAFT) surrogate data (e.g. Supplementary Fig. 1c). AAFT surrogates are designed to preserve both the frequencies (i.e. autocorrelations and power spectra) and amplitudes (i.e. correlations and/or noise) of the original data, but to break any causal coupling by randomizing the phases of the frequency components[9,10]. Significant IT is assessed by comparison with a distribution of IT values calculated from a large number (e.g. 10^4) of pairs of surrogate time series.

Directional IT analysis between two time series (e.g. R and O) involves three significance tests (Supplementary Fig. 2): (1) Is the directional IT from R to O significant (Supplementary Fig. 2a, d)? (2) Is the directional IT from O to R significant (Supplementary Fig. 2b, e)? (3) If one or both, then is the IT in one direction significantly greater than in the opposite direction (Supplementary Fig. 2c, f)? Note that the IT varies as a function of the bin size used for gridding the data, but for the significance test, we integrate across bin sizes, using the area under the curve as an informal measure of the total IT[6]. In this example, we find that IT is significant in both directions, but R→O is significantly greater than O→R (denoted R>O), representing a significantly asymmetric, yet bidirectional information flow. This result agrees with the intuitive expectation that

the obstructed breathing pattern drives the oxygen concentration, but that the breathing intermittently responds to low oxygen levels via feedback mechanisms.

However, the results described above (Supplementary Fig. 2) represent a single, random sampling of the system, and we would like to know how robust this result is to the sampling. A sensitivity analysis is performed by varying the number of time steps sampled, *N*, over a relevant range (in this case from *N*=10 to *N*=150 samples). For each value of *N*, the original time series (Supplementary Fig. 1a) are iteratively sampled (500 iterations) by selecting *N* uniformly random time steps. In each iteration, the three IT significance tests (Supplementary Fig. 2) are performed on the sampled data. The sampling robustness of the IT can then be evaluated by tracking the proportion of significant results (frequency of detection, or statistical sensitivity) for each value of *N* (Supplementary Fig. 3). Note that because the values are frequencies, the third significance test is plotted in both directions (R>O and O>R, although these are mutually exclusive in a single analysis.

As sampling approaches 100 time steps, IT between R and O becomes invariably significant, in both directions (Supplementary Fig. 3), suggesting a two-way interaction. However, significant R>O is detected with increasing frequency, indicating a dominant directionality of information flow, consistent with the interpretation given above. Spearman rank-order correlation on first differences is rarely significant (Supplementary Fig. 3), because the relationship is not linear or monotonic.

Still, the relationship between R and O might be spurious, if both were driven by a third variable, such as H. This is tested by conditioning the IT on a third variable (analogous to a partial correlation): is the IT between R and O still significant when we take into account any common interaction with H (O ← R | H)? The answer is yes (Supplementary Fig. 4), implying that R contains information (not found in H) that is useful for predicting changes in O. Conditional IT (CIT) from H to O also approaches significance beyond mutual interaction with R (O ← H | R), suggesting that both R and H contain information useful for predicting O, but the former is typically significantly stronger (to reduce clutter, the significance of the difference is not plotted here). Partial correlations on first differences give the opposite result in this case (Supplementary Fig. 4).

Because of the non-symmetric nature of the IT, the pairwise IT results may be required to interpret the CIT: If there is some asymmetry (not necessarily significant) favouring Y→X over X→Y, then, even if pairwise Z→X and Z→Y are equal, Y←Z | X will be greater than X←Z | Y (i.e. Y will be a stronger conditioning variable than X). We refer to this effect when describing CIT results in the main paper (see Results section).

This example is instructive for several reasons: (i) cardiorespiratory physiology is a complex system near to one's heart, with multiple interacting components, non-linearity, and feedbacks; (ii) IT has the potential to quantify relative strength and directionality of coupling without recourse to

mechanistic model assumptions; (iii) standard correlations are of little use or potentially misleading in this case; (iv) IT requires only relative age control, as is often the case with stratigraphic data; (v) IT can detect non-trivial interactions with relatively sparse data.

The use of AAFT surrogates helps avoid false positive results that may stem from frequency bias in the IT[7,10]. However, when analyzing a pair of time series, the IT may still be sensitive to large differences in the degree of non-stationary in the two time series. To evaluate whether or not differences in non-stationarity could be a contributing factor to the directional asymmetry, we used the KPSS test[11] to calculate a test score (1 if the null hypothesis of stationarity is rejected, 0 if not) over all possible lags *K*. A bias index was defined as the sum of the absolute values of the difference between the two KPSS test score vectors, divided by *K* (a similar index was proposed in Hannisdal et al.[12]). Thus, a maximum bias value of 1 means that one time series is stationary at all lags, whereas the other time series is non-stationary at all lags, representing a maximum possible bias. Conversely, a bias value of 0 means that both series are either fully stationary, or non-stationary at the exact same lags, representing a minimum possible bias.

To reduce non-stationarity, all time series are pre-processed by detrending (subtracting a best-fit polynomial or spline), and power transformation (log, or Box-Cox). The data are also normalized (mean = 0, standard deviation = 1), which enables quantitative comparison of IT in both directions, and allows the data gridding bin size to be conveniently expressed in units of standard deviation (Supplementary Fig. 2a-c). The effectiveness of data pre-processing can be illustrated with random walks (red noise, or AR(1) processes), which are typically highly non-stationary (Supplementary Fig. 5). Even after pre-processing, some differences remain (e.g. red stippled line in Supplementary Fig. 5b), and these are typically present also in the surrogates, but a bias value of ~0.1 or lower has not been found to induce spurious asymmetry in the IT.

4. Supplementary References

1. Rigney, D. R. *et al.* in *Time series prediction: forecasting the future and understanding the past* (eds A.S. Weigend & N.A. Gershenfeld) 105-129 (Addison-Wesley, 1993).
2. Prokoph, A., Shields, G. A. & Veizer, J. Compilation and time-series analysis of a marine carbonate $\delta^{18}O$, $\delta^{13}C$, $^{87}Sr/^{86}Sr$ and $\delta^{34}S$ database through Earth history. *Earth-Science Reviews* **87**, 113-133, doi:http://dx.doi.org/10.1016/j.earscirev.2007.12.003 (2008).
3. Cardenás, A. L. & Harries, P. J. Effect of nutrient availability on marine origination rates throughout the Phanerozoic eon. *Nature Geoscience* **3**, 430-434, doi:10.1038/ngeo869 (2010).
4. Benjamini, Y. & Hochberg, Y. Controlling the false discovery rate: a practical and powerful approach to multiple testing. *Journal of the Royal Statistical Society, Series B* **57**, 289-300 (1995).
5. Schreiber, T. Measuring information transfer. *Physical Review Letters* **85**, 461-464 (2000).
6. Verdes, P. F. Assessing causality from multivariate time series. *Physical Review E* **72**, 026222 (2005).
7. Hannisdal, B. Non-parametric inference of causal interactions from geological records. *American Journal of Science* **311**, 315-334, doi:10.2475/04.2011.02 (2011).
8. Hannisdal, B. Detecting common-cause relationships with directional information transfer. *Geological Society, London, Special Publications* **358**, 19-29, doi:10.1144/sp358.3 (2011).
9. Schreiber, T. & Schmitz, A. Surrogate time series. *Physica D-Nonlinear Phenomena* **142**, 346-382 (2000).
10. Vejmelka, M. & Paluš, M. Inferring the directionality of coupling with conditional mutual information. *Physical Review E* **77**, 026214 (2008).
11. Kwiatkowski, D., Phillips, P. C. B. & Schmidt, P. Testing the null hypothesis of stationarity against the alternative of a unit root. *Journal of Econometrics* **54**, 159-178 (1992).
12. Hannisdal, B., Henderiks, J. & Liow, L. H. Long-term evolutionary and ecological responses of calcifying phytoplankton to changes in atmospheric CO_2. *Global Change Biology* **18**, 3504-3516 (2012).
